# Interleukin (IL)-6 Inhibits IL-27- and IL-30-Mediated Inflammatory Responses in Human Monocytes

**DOI:** 10.3389/fimmu.2018.00256

**Published:** 2018-02-15

**Authors:** Carlene Petes, Mélissa K. Mariani, Yawen Yang, Nathalie Grandvaux, Katrina Gee

**Affiliations:** ^1^Department of Biomedical and Molecular Sciences, Queen’s University, Kingston, ON, Canada; ^2^Centre de Recherche du CHUM (CRCHUM), Université de Montréal, Montréal, QC, Canada

**Keywords:** interleukin-30, interleukin-27, inflammation, human monocytes, human CD4 T cells

## Abstract

Interleukin (IL)-30, the IL-27p28 subunit of the heterodimeric cytokine IL-27, acts as an antagonist of IL-27 and IL-6 signaling in murine cells via glycoprotein 130 (gp130) receptor and additional binding partners. Thus far, functions of IL-30 have not been fully elucidated in human cells. We demonstrate that like IL-27, IL-30 upregulated TLR4 expression to enhance lipopolysaccharide-induced TNF-α production in human monocytes; however, these IL-30-mediated activities did not reach the same levels of cytokine induction compared to IL-27. Interestingly, IL-30- and IL-27-mediated interferon-γ-induced protein 10 (IP-10) production required WSX-1 engagement and signal transducer and activator of transcription (STAT) 3 phosphorylation; furthermore, IL-30 induced STAT phosphorylation after 16 h, whereas IL-27 induced STAT phosphorylation within 30 min. This prompted us to examine if a secondary mediator was required for IL-30-induced pro-inflammatory functions, and hence we examined IL-6-related molecules. Combined with inhibition of soluble IL-6 receptor α (sIL-6Rα) and data showing that IL-6 inhibited IL-30/IL-27-induced IP-10 expression, we demonstrate a role for sIL-6Rα and gp130 in IL-30-mediated activity in human cells.

## Introduction

Interleukin (IL)-27, discovered by Pflanz et al., is composed of non-covalently associated IL-27p28 helical subunit with Epstein–Barr virus (EBV)-induced gene 3 (EBI3)-soluble receptor subunit (1). This heterodimeric cytokine is produced by activated antigen-presenting cells such as monocytes, macrophages, and dendritic cells (DCs) (1, 2). The IL-27p28 subunit alone is called IL-30 and functions in the absence of EBI3 (3–5). IL-27p28 is produced in response to TLR agonists such as lipopolysaccharide (LPS) or CpG oligodeoxynucleotides as well as interferon (IFN)-γ treatment of murine and human monocytic cells (5–7). IL-27 signals through the heterodimeric receptor subunits WSX-1 (IL-27 receptor α/T cell cytokine receptor) and glycoprotein 130 (gp130) to induce activation of signal transducer and activator of transcription (STAT) 1 and STAT3 in monocytic cells (2, 8). WSX-1 and gp130 are found co-expressed on monocytes, macrophages, DCs, T and B lymphocytes, natural killer cells, mast cells, and endothelial cells (2, 9, 10), allowing these cells to respond to IL-27 and possibly IL-30.

The role of IL-30 in innate immunity has been recently established although most of the studies on IL-30 signaling have been developed in murine models (3–5). It has been suggested that IL-30 requires a binding partner to signal through gp130; for example, cytokine-like factor (CLF)-1 or soluble IL-6 receptor α (sIL-6Rα) have been shown to interact with IL-30 (3, 5, 11). In murine EBI3-deficient (EBI3^−/−^) splenocytes, pretreatment of IL-27p28 antagonizes IL-6, IL-11, and IL-27 signaling by binding the immunoglobulin (IgG)-like domain of gp130 (5). By using CD4 T cells from WSX-1-deficient (WSX-1^−/−^) mice, distinct receptor requirements for IL-27- and IL-30-induced STAT3 phosphorylation were determined. IL-27-stimulated WSX-1^−/−^ CD4 T cells exhibited less STAT3 phosphorylation relative to wild type, while IL-30 induced STAT3 phosphorylation equally in WSX-1^−/−^ and wild-type cells (11). However, stimulation with IL-30, but not IL-27, was capable of inducing STAT3 phosphorylation in WSX-1^−/−^ NIH3T3 cells upon transfection with IL-6Rα (11). Knowledge so far regarding IL-30 receptor engagement suggests a dependency on additional binding partners for optimal signaling through gp130 and/or WSX-1; nonetheless, how IL-30 signals in human immune cells has not been fully characterized.

Our study demonstrates that IL-30 signals as an independent cytokine with comparable functions to IL-27 in human monocytic cells with regards to the characteristic production of chemokine IFN-γ-inducible protein 10 (IP-10; CXCL10). Furthermore, like IL-27 (12), IL-30 enhanced TLR4 expression in human monocytes, priming cells for greater LPS responsiveness. Examination of IL-27 and IL-30 signal transduction in human monocytes revealed that both STAT3 and STAT1 were phosphorylated; however, the IL-27-induced activation of these molecules was significantly greater than that of IL-30, which was very weak at early time points. Further analysis revealed that significant STAT phosphorylation occurred after 16 h in response to IL-30 as well as a secondary wave of IL-27-induced STAT activation. Interestingly, engagement of WSX-1 is required for IL-30 signaling and downstream IP-10 production in human monocytes, while murine models have proposed that WSX-1 is not involved in IL-30 signaling (11, 13). By using the metalloproteinase inhibitor, BB-94, a requirement for sIL-6Rα in IL-30 and IL-27 functions was observed in human monocytic cells. Alternatively, addition of IL-6, which binds IL-6Rα (or sIL-6Rα) and gp130, inhibited IL-30- and IL-27-induced IP-10 production. This study identifies the WSX-1 and sIL-6Rα receptor requirements for optimal IL-30 responsiveness in human monocytic cells and provides evidence that IL-30 may have overlapping functions with IL-27 in human innate immunity.

## Materials and Methods

### Cell Lines, Cell Culture, and Reagents

**Figure 1:**
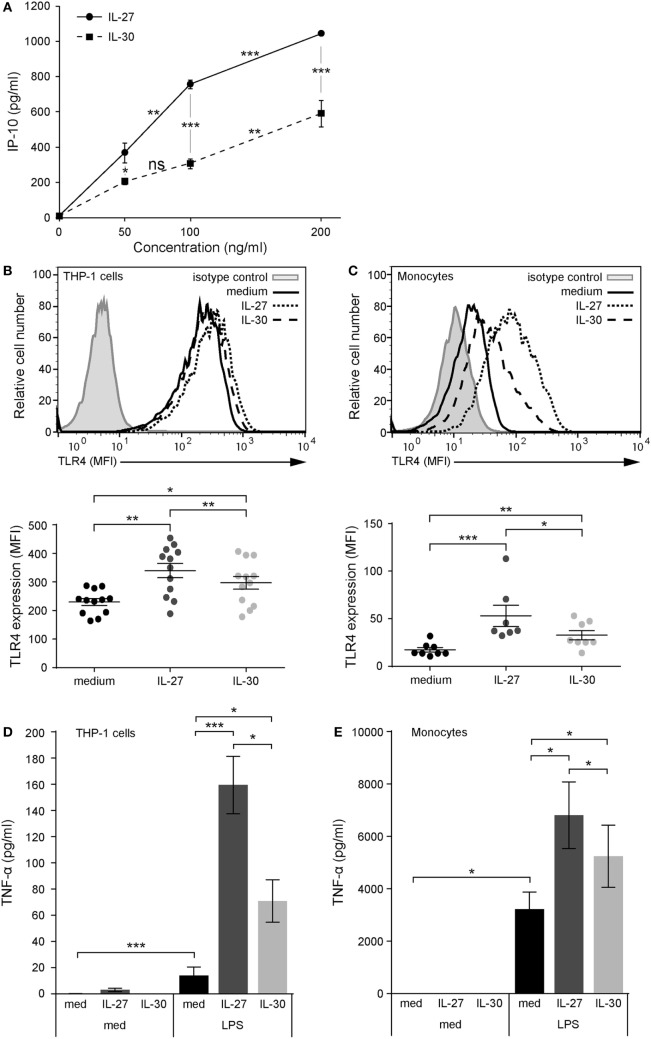
IL-30 and IL-27 prime cells for enhanced lipopolysaccharide (LPS) responsiveness via TLR4 upregulation. **(A)** THP-1 cells were treated with IL-27 or IL-30 at 50, 100, or 200 ng/mL for 24 h. IP-10 production was measured in cell-free supernatants by ELISA. Data presented are the mean ± SD of four different THP-1 technical replicate wells. Mann–Whitney *U* tests were used for statistical analyses between groups as indicated. **(B)** THP-1 cells and **(C)** primary human monocytes were stimulated with or without recombinant IL-27 (50 ng/mL) or IL-30 (50 ng/mL) for 24 h. TLR4 expression was measured using flow cytometry, and representative histograms are shown (top). Mean fluorescence intensity corresponding to TLR4 expression was measured for each different THP-1 experiment and different monocyte donors. Data presented include mean ± SEM of all data points (bottom). **(D)** THP-1 cells and **(E)** primary human monocytes were treated with or without recombinant IL-27 (50 ng/mL) or IL-30 (50 ng/mL) for 16 h and then washed and stimulated with LPS (1 µg/mL) for 4 h. TNF-α production was measured in cell-free supernatants by ELISA. Data presented are the mean ± SEM of eight different THP-1 experiments or six different monocyte donors. Mann–Whitney *U* tests were used for statistical analyses between medium and IL-27/IL-30. Wilcoxon matched-pairs signed-rank test was used for statistical analyses between IL-27 and IL-30. *p ≤ 0.05; **p ≤ 0.01; ***p ≤ 0.001.

The THP-1 human promonocytic cell line was purchased from the American Type Culture Collection. Cells were cultured in Gibco RPMI (Life Technologies, Burlington, ON, Canada) supplemented with 10% Fetal Bovine Serum (FBS; Hyclone, Logan, UT). Recombinant human IL-27 and IL-6 and recombinant murine IL-27p28 were purchased from R&D Systems (Minneapolis, MN, USA). As outlined on the manufacturer’s product sheets, biological activity of rhIL-27 (50 ng/mL) was measured for antiviral activity using human HepG2 cells infected with encephalomyocarditis virus, biological activity of rhIL-6 was measured for cell proliferation of mouse T1165.85.2.1 cells, and biological activity of rmIL-27p28 was measured for its ability to induce IP-10 secretion by THP-1 cells. Upon analysis of IL-27 and IL-30 dose responses (50–200 ng/mL), we observed that IL-27-induced IP-10 expression was significantly higher than that for IL-30-induced IP-10 expression at all concentrations tested (Figure 1A); therefore, we used doses of 50 ng/mL for each cytokine for our experiments. Neutralizing antibodies (nAbs) for human IL-27, IL-30, and gp130; WSX-1 Fc chimera; and mouse IgG1 and human IgG1 isotype controls were also purchased from R&D systems. Other reagents used include Stattic (Tocris Bioscience, Bristol, UK), Batimastat (BB-94) (EMD Millipore, Burlington, MA, USA), and solvent control DMSO (Bioshop, Burlington, ON, Canada). Recombinant human IL-6Rα was purchased from BioLegend (San Diego, CA, USA).

### Human Primary Monocyte Isolation

Whole blood was drawn from healthy volunteers obtained in accordance with the recommendations of Canadian Tri-Council Policy Statement: Ethical Conduct for Research Involving Humans and Health Sciences and Affiliated Teaching Hospitals Research Ethics Board (HSREB) with written informed consent from all subjects. All subjects gave written informed consent in accordance with the Declaration of Helsinki. The protocol was approved by the HSREB. Primary human monocytes were isolated using RosetteSep™ negative selection human monocyte enrichment cocktail (Stem Cell Technologies, Vancouver, BC, Canada) according to manufacturer’s instructions. Briefly, whole blood was supplemented with respective enrichment cocktails and layered over top of the density gradient Lympholyte-H separation medium (Cedarlane, Ballintemple, Ireland) in SepMate 50-mL conical tubes (StemCell Technologies). Buffy coat was extracted after centrifugation, and cells were washed with PBS + 10 mM EDTA + 2% FBS. Cells were incubated in Gibco RPMI 1640 and supplemented with 10% FBS at 37°C and 5% CO_2_ and cultured in the presence or absence of IL-30 or IL-27 for the time points described.

### ELISA

Culture supernatants were used to quantify cytokine expression according to manufacturers’ instructions for human TNF-α (Thermo Fisher Scientific, Affymetrix eBioscience, Waltham, MA, USA; Ready-Set-Go kit; sensitivity: 4–500 pg/mL), human CXCL10/IP-10 (R&D Systems, DuoSet kit; sensitivity: 31.2–2,000 pg/mL), and sIL-6Rα (R&D systems, DuoSet kit; sensitivity: 15.6–1,000 pg/mL). All supernatants were either undiluted or diluted 1:2 and 1:5 and were run on the same plate to ensure the readings were comparable. Absorbance was measured with the BioTek ELx800 Microplate Reader (Winooski, VT, USA) at 450 nm. Graphic data present the mean ± SEM, and *n* values for each experiment are indicated in figure legends.

### Flow Cytometry

For surface staining, THP-1 cells and primary monocytes were resuspended in FACS buffer (PBS + 0.01% sodium azide + 2% FBS) and incubated with anti-human TLR4 AlexaFluor^®^ 488 (eBioscience), anti-human WSX-1 FITC (R&D Systems), or anti-human gp130 PE (R&D Systems). Cells incubated with anti-human CD126 (IL-6Rα) antibody (eBioscience) were subsequently stained with anti-mouse PE secondary antibody (BioLegend). Mouse IgG2a κ FITC and PE isotype controls (eBioscience) were used at the same concentration and dye:protein ratio as the TLR4, WSX-1, gp130, and IL-6Rα antibodies. Secondary antibody was added alone as a control. Primary monocytes were stained with CD14 Biotin (eBioscience) and streptavidin AlexaFluor^®^ 610-R-phycoerythrin conjugate (Invitrogen, Carlsbad, CA, USA) to assess population purity, which was found to be greater than 95%. CD14^+^ primary cells were gated on for analysis of TLR4, WSX-1, gp130, or IL-6Rα. Data were acquired with the Epics XLMCL or CytoFLEX flow cytometer (Beckman Coulter, Pasadena, CA, USA) and analyzed using FlowJo software, version X 10.0.7r2.

### Immunoblot Analysis

Cell pellets were lysed on ice using Nonidet P-40 lysis buffer as previously described (14). Whole-cell extracts were quantified using the Bradford protein assay (Bio-Rad, Hercules, CA, USA), resolved by SDS-PAGE, and transferred to nitrocellulose membrane before analysis by immunoblot. Membranes were blocked in PBS containing 0.5% Tween (PBS-T) and 5% non-fat dry milk before incubation with the following primary antibodies: anti-actin (Cat #MAB1501, Millipore, Billerica, MA, USA), anti-tubulin (B-7, Cat #sc-5286, Santa-Cruz Biotechnology, Dallas, TX, USA) and anti-STAT1-phospho-Tyr701 (Cat #9171), anti-STAT3-phospho-Tyr705 (Cat #9145), anti-STAT1 (Cat #9172), and anti-STAT3 (Cat #4904) all from Cell Signaling Technology (Danvers, MA, USA). After washes in PBS-T, membranes were further incubated with horseradish peroxidase-conjugated secondary antibodies (KPL, Milford, MA, USA, or Jackson Immunoresearch Laboratories, West Grove, PA, USA). Antibodies were diluted in PBS containing 0.5% Tween and either 5% non-fat dry milk or BSA. After washes in PBS-T, immunoreactive bands were visualized by enhanced chemiluminescence (Western Lightning Chemiluminescence Reagent Plus, Perkin-Elmer Life Sciences, Waltham, MA, USA) using a LAS4000mini CCD camera apparatus (GE Healthcare, Little Chalfont, UK).

### Statistical Analysis

Statistical analyses were performed with GraphPad Prism 6. For all data sets, *p* values were calculated using Mann–Whitney *U* test and Wilcoxon matched-pairs signed-rank test. A *p* value less than 0.05 was used to define statistical significance. Data are represented as the cumulative mean ± SEM of biological replicates, and *n* values for each experiment are indicated in the figure legends.

## Results

### IL-30 Enhances TLR4 and Subsequent LPS-Induced TNF-α Production

To model IL-27 and IL-30 functions, we focused on human monocytic cells using the THP-1 cell line and primary monocytes as model systems. We previously reported that IL-27 mediates pro-inflammatory cytokine and chemokine production in human monocytes, including IP-10, thus we decided to use this chemokine as a readout for dose–response analysis (8). THP-1 cells were treated with increasing doses of IL-30 or IL-27 for 24 h, and IP-10 expression was assessed. Both cytokines induced significant IP-10 expression at 50 ng/mL (Figure 1A); thus, we chose this dose for subsequent experiments. Previously, our lab demonstrated that IL-27 upregulates TLR4 expression and LPS responsiveness in human monocytes (12). Therefore, to investigate the role of IL-30 in modulating TLR4 expression, we treated THP-1 cells and primary human monocytes with IL-30 (50 ng/mL; 2 nM) or, as a positive control, IL-27 (50 ng/mL; 1 nM) for 24 h and quantified TLR4 expression levels by flow cytometry (Figures 1B,C). IL-30 significantly enhanced TLR4 expression on THP-1 cells (Figure 1B) and primary monocytes (Figure 1C) although to a significantly lesser extent to that of IL-27. To investigate whether IL-30-induced TLR4 is sufficient to prime cells for heightened LPS responses, THP-1 cells and primary human monocytes were pretreated with medium, IL-27, or IL-30 for 16 h and subsequently stimulated with LPS (1 µg/mL) for an additional 4 h. IL-30 pretreatment of THP-1 cells (Figure 1D) and primary human monocytes (Figure 1E) enhanced LPS-induced TNF-α production compared to LPS alone. As expected, IL-27 pretreatment upregulated TLR4 expression and LPS-induced TNF-α expression, but this was to a significantly greater degree than IL-30. Taken together, these results suggest that IL-30 shares functions with IL-27 in human monocytic cells; however, IL-30-mediated effects were not as potent as those with IL-27.

### IL-30 Induces IP-10 Production and Functions Independently of IL-27

**Figure 2:**
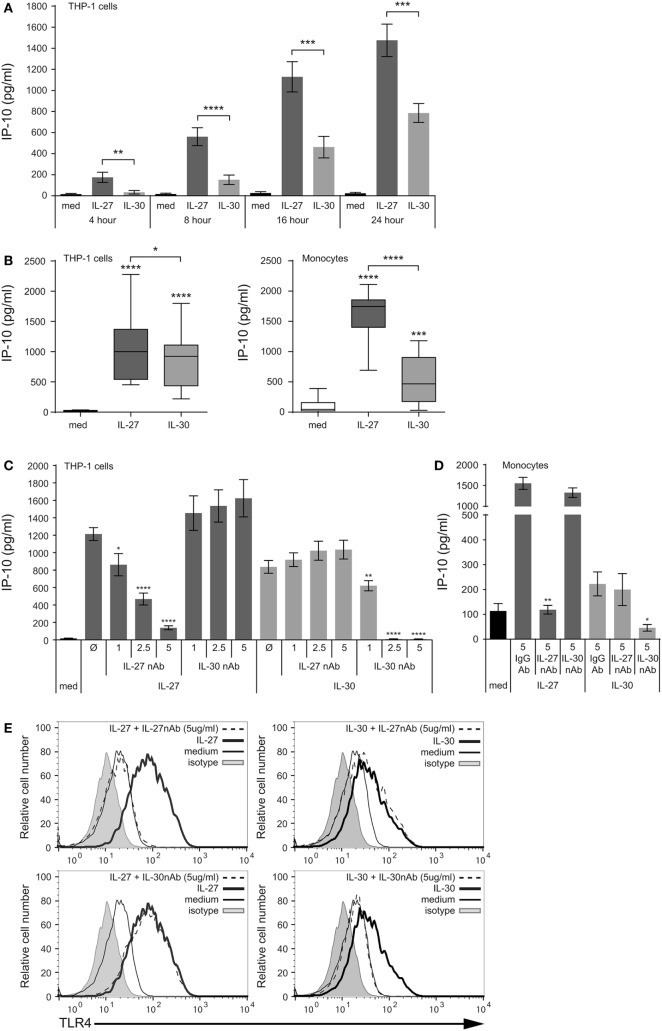
IL-30 induces IP-10 production and functions independently of IL-27. **(A)** THP-1 cells were stimulated with or without recombinant IL-27 (50 ng/mL) or IL-30 (50 ng/mL) for 4, 8, 16, and 24 h. IP-10 production was measured in cell-free supernatants by ELISA. Data are the mean ± SEM of at least seven different THP-1 experiments. **(B)** THP-1 cells (left) and primary human monocytes (right) were treated with recombinant IL-27 (50 ng/mL) or IL-30 (50 ng/mL) for 24 h. IP-10 production was measured in cell-free supernatants. Box and whisker plots include all data points from at least 10 different THP-1 experiments and 15 different monocyte donors. **(C)** THP-1 cells and **(D)** primary human monocytes were treated with or without recombinant IL-27 (50 ng/mL) or IL-30 (50 ng/mL) in the presence of neutralizing antibodies (nAbs) for IL-27 (1–5 µg/mL as indicated) or IL-30 (1–5 µg/mL as indicated) for 24 h. IP-10 production was measured in cell-free supernatants by ELISA. Data presented are the mean ± SEM of three different THP-1 experiments and four different monocyte donors. **(E)** Primary human monocytes were stained with TLR4-AlexaFluor488 antibody, and TLR4 expression was measured using flow cytometry. Histograms are representative of at least four different monocyte donors. Mann–Whitney *U* tests were used for statistical analyses between medium control (med) and IL-27/IL-30 **(B)** and between IL-27 or IL-30 control (Ø) and nAb treatments **(C)**; or as indicated by brackets **(A,B)**. *p ≤ 0.05; **p ≤ 0.01; ***p ≤ 0.001; ****p ≤ 0.0001.

To investigate the signaling mechanisms involved in the function of IL-30 and IL-27 in human monocytes, we further assessed IP-10 production in response to IL-30 and IL-27. Initially, THP-1 cells were cultured in the presence or absence of IL-27 (50 ng/mL; 1 nM) or IL-30 (50 ng/mL; 2 nM) for 4–24 h, and IP-10 secretion was quantified in cell-free supernatants by ELISA (Figure 2A). Both IL-27 and IL-30 induced IP-10 secretion; however, IL-30 produced significantly less IP-10 compared to IL-27 at all time points measured. Of note, IL-30 stimulation did not induce TNF-α and IL-6 production in THP-1 cells (data not shown), two cytokines previously demonstrated to be produced in response to IL-27 in primary monocytes (8). We then compared IL-30- and IL-27-mediated IP-10 production from THP-1 cells and primary human monocytes after 24 h. Both IL-30 and IL-27 induced IP-10 secretion; however, we observed a range in responsiveness to each cytokine, notably in primary human monocytes where some donors exhibited very low responses to IL-30 (Figure 2B). Box and whisker plots including all outliers depict the range of IL-27- and IL-30-induced IP-10 expression of at least 10 independent experiments for both THP-1 cells and primary human monocytes. Basal IP-10 expression levels in THP-1 cells ranged from undetectable to 35.9 pg/mL, while in primary cells, basal expression was slightly higher, ranging from undetectable to 390.0 pg/mL. The detected concentrations of IL-30-induced IP-10 ranged from 30.9 to 1180.7 pg/mL in primary monocytes and 219.0 to 1798.8 pg/mL in THP-1 cells. On the other hand, IL-27 consistently induced IP-10 at significantly higher concentrations in each cell type, ranging from 693.0 to 2112.4 pg/mL in primary monocytes and 452.8 to 2277.6 pg/mL in THP-1 cells. These results confirm our observation from Figure 1 that IL-30-mediated functions in human monocytic cells are significantly lower than those induced by IL-27.

Since IL-30 is a constituent of IL-27 as the IL-27p28 subunit, we sought to determine if this subunit is the key component required for the induction of IL-27-mediated responses. Therefore, to determine if IL-30 and IL-27 function independently, we used specific nAbs to IL-27 and IL-30. Cells were cultured in medium alone, IL-27 (50 ng/mL), or IL-30 (50 ng/mL) in the presence or absence of IL-27 nAb (1–5 µg/mL) or IL-30 nAb (1–5 µg/mL) for 24 h (Figures 2C–E). We observed a dose-dependent inhibition of IL-27-induced IP-10 production by the IL-27 nAb but not by the IL-30 nAb in THP-1 cells (Figure 2C). As for IL-30, while its function was abrogated by IL-30 neutralization, it was not significantly inhibited by the IL-27 nAb in THP-1 cells (Figure 2C). This trend was recapitulated in primary human monocytes using the highest doses of the IL-27 nAb and IL-30 nAb (Figure 2D). This indicates that even though IL-27p28 is a component of IL-27, the IL-27p28 (IL-30) nAb is specific to epitopes on IL-30 and not IL-27. Furthermore, the two nAbs act as controls for one another, since they are polyclonal goat IgG; the IL-30 nAb does not impact IL-27-induced IP-10 and the IL-27 nAb does not impact IL-30-induced IP-10. In addition, isotype control goat IgG antibody did not have an effect on IL-27- or IL-30-induced IP-10 production in monocytes. To further confirm that neutralization of the IL-27p28 subunit does not impact IL-27-mediated functions, we stimulated primary human monocytes with IL-27 or IL-30 in the presence of each of the two nAb. The data show that IL-27- and IL-30-mediated TLR4 upregulation is abolished by the IL-27 nAb and IL-30 nAb, respectively (Figure 2E), confirming our above observations in Figures 2C,D. This demonstrates that IL-30 functions independently of IL-27 with respect to pro-inflammatory responses in human monocytes.

### Short-term IL-30 Stimulation Induces Weak Phosphorylation of STAT3 and STAT1

**Figure 3:**
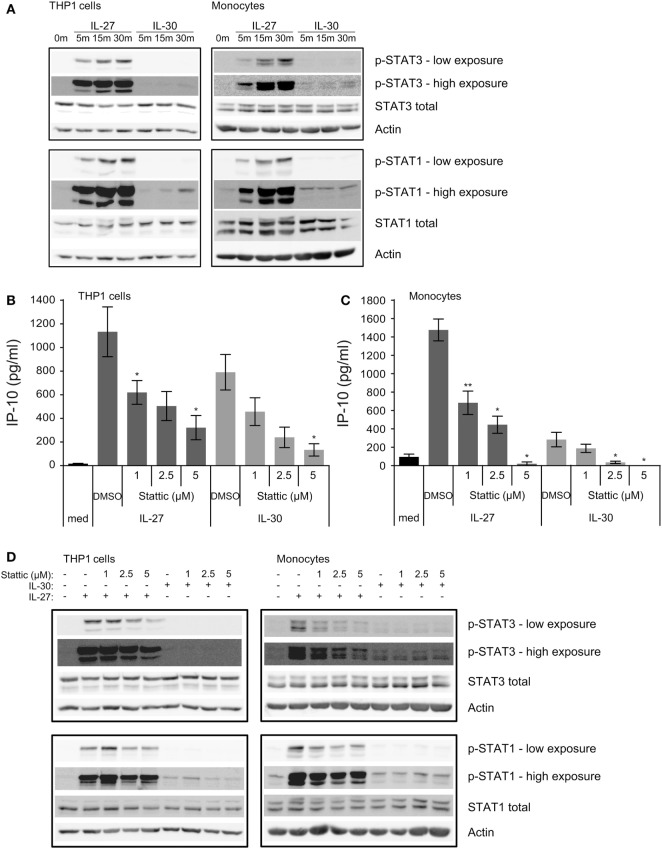
IL-30 and IL-27 differentially activate STAT3 and STAT1. **(A)** THP-1 cells and primary human monocytes were treated with or without recombinant IL-27 (50 ng/mL) or IL-30 (50 ng/mL) for 5, 15, or 30 min and STAT3 and STAT1 phosphorylation was measured by immunoblot. Blots were stripped and reprobed for total STAT3 and STAT1 levels as well as β-actin as a loading control. Blots are representative of three different THP-1 experiments or three different monocyte donors. **(B,D)** THP-1 cells and **(C,D)** primary human monocytes were treated with or without recombinant IL-27 (50 ng/mL) or IL-30 (50 ng/mL) in the presence or absence of Stattic (1–5 µM as indicated) or DMSO solvent control for **(B,C)** 24 h or **(D)** 30 min. **(B,C)** IP-10 production was measured in cell-free supernatants by ELISA. Data presented are the mean ± SEM of three different THP-1 experiments or three different monocyte donors. Mann–Whitney *U* tests were used for statistical analyses between respective IL-27 or IL-30 control (DMSO) and Stattic treatments. *p ≤ 0.05; **p ≤ 0.01. **(D)** Phosphorylation of STAT3 and STAT1 were measured by immunoblot. Total STAT3 and STAT1 levels were used as controls, and β-actin was used as a loading control. Blots are representative of three different THP-1 experiments or three different monocyte donors.

Murine models suggest that IL-30 solely signals through the gp130-STAT3 axis (11), whereas IL-27 induces the phosphorylation of STAT3 and STAT1 in human monocytes (8); therefore, we investigated whether IL-30 engages STAT3 as well as STAT1 in human monocytic cells. We stimulated THP-1 cells and primary human monocytes with IL-27 or IL-30 for 5–30 min and examined STAT3 and STAT1 phosphorylation by immunoblot (Figure 3A). As expected, IL-27 highly activated STAT3 and STAT1 within 5 min and increased over time, peaking at 30 min. In contrast, IL-30 induced only minimal STAT1 phosphorylation compared to IL-27, and IL-30-induced STAT3 phosphorylation was barely detectable after 30 min in monocytes and THP-1 cells.

Given that much of the literature regarding IL-30 signaling indicates the involvement of the gp130-STAT3 signaling axis (5, 11), we investigated whether inhibition of STAT3 activation could modulate IL-30 responses in human monocytes. Therefore, we used Stattic, a small molecule that blocks STAT3 activation and dimerization via inhibition of tyrosine-phosphorylated binding motifs from binding the STAT3 Src homology 2 domains (15). We cultured THP-1 cells and primary human monocytes in medium alone or in the presence of IL-27 or IL-30 with or without Stattic (1–5 µM) or DMSO (solvent control) for 24 h and measured IP-10 production by ELISA (Figures 3B,C) and STAT3 and STAT1 phosphorylation after 30 min by immunoblot (Figure 3D). Interestingly, Stattic inhibited both IL-27- and IL-30-induced IP-10 production in a dose-dependent manner, with statistically significantly reduction in THP-1 cells and complete abrogation of IP-10 production in primary monocytes at 5 µM of Stattic compared to DMSO solvent controls (Figures 3B,C). Immunoblot analysis showed that Stattic inhibited IL-27-induced STAT3 phosphorylation and not STAT1 phosphorylation in THP-1 cells, while in primary monocytes, Stattic inhibited IL-27-induced STAT3 phosphorylation and partially affected that of STAT1 (Figure 3D). The partial STAT1 inhibition may be due to non-specific effects of Stattic on STAT1 as others have documented (16). Stattic did not notably reduce IL-30-induced STAT phosphorylation in either cell type. Overall, the data indicate a requirement for STAT3 activation in IL-30-induced IP-10 production after 24 h.

### Engagement of the WSX-1 Receptor Is Required for IL-30-Induced IP-10 Production and STAT Signaling

**Figure 4:**
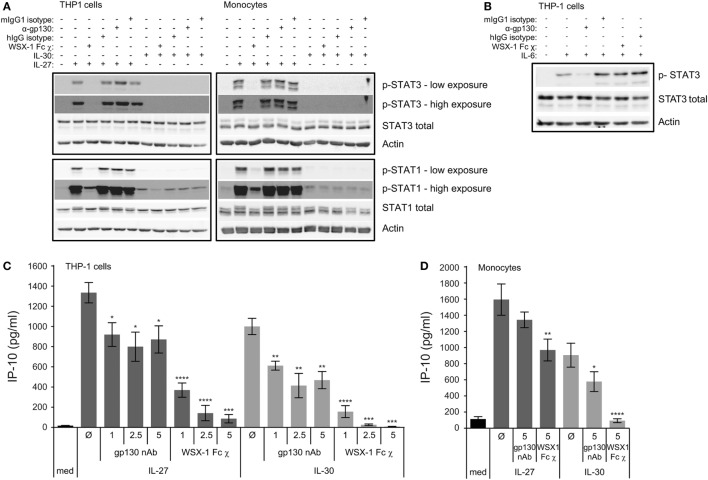
Engagement of WSX-1 is required for IL-30-IP-10 production and STAT activation. THP-1 cells and primary human monocytes were treated with or without **(A)** recombinant IL-27 (50 ng/mL) or IL-30 (50 ng/mL) or **(B)** recombinant IL-6 (10 ng/mL) in the presence or absence of WSX-1 Fc chimera (χ) (1 µg/mL), human IgG isotype as a chimera control (1 µg/mL), anti-glycoprotein 130 (gp130; 1 µg/mL), or murine IgG1 isotype as an anti-gp130 control (1 µg/mL) for 30 min. Phosphorylation of STAT3 and STAT1 and total STAT3 and STAT1 was measured by immunoblot, and β-actin was used as a loading control. Blots are representative of three different THP-1 experiments or three different monocyte donors. **(C)** THP-1 cells and **(D)** primary human monocytes were treated with or without recombinant IL-27 (50 ng/mL) or IL-30 (50 ng/mL) in the presence or absence of WSX-1 Fc χ (1–5 µg/mL as indicated) or anti-gp130 (1–5 µg/mL as indicated) for 24 h. IP-10 production was measured in cell-free supernatants by ELISA. Data presented are the mean ± SEM of at least four different THP-1 experiments or four different monocyte donors. Mann–Whitney *U* tests were used for statistical analyses between respective IL-27 or IL-30 control (Ø) and gp130 neutralizing antibody (nAb) or WSX-1 Fc χ. *p ≤ 0.05; **p ≤ 0.01; ***p ≤ 0.001; ****p ≤ 0.0001.

The indication that IL-30 may follow a differential signaling pathway from IL-27, as demonstrated in Figure 3A, led us to investigate the IL-30 receptor requirements. Given that IL-27 signaling requires both WSX-1 and gp130 (2), and IL-30 signaling appears to require gp130 (5, 11), we investigated these receptors. We inhibited IL-27 and IL-30 binding to either gp130 or WSX-1 receptor chains using gp130 nAb or a WSX-1 Fc chimera, respectively. THP-1 cells and primary human monocytes were cultured in medium, IL-27, or IL-30 in the presence or absence of gp130 nAb (1–5 µg/mL), WSX-1 Fc chimera (1–5 µg/mL) or appropriate isotype controls. To demonstrate receptor requirements for signaling, STAT3 and STAT1 phosphorylation were measured by immunoblot after 30 min (Figure 4A). Phosphorylation of STAT1 following IL-27 and IL-30 treatment as well as IL-27-induced STAT3 phosphorylation were inhibited by WSX-1 Fc chimera (1 µg/mL) compared to the human IgG isotype control. This suggests that binding to WSX-1 is required for IL-30-induced JAK/STAT signaling. Surprisingly, neutralization of gp130 did not reduce IL-27- or IL-30-induced STAT3 or STAT1 phosphorylation. To confirm the specificity of the gp130 nAb and WSX-1 Fc chimera, we stimulated THP-1 cells in the presence or absence of the gp130 nAb or the WSX-1 Fc chimera with recombinant human IL-6 (10 ng/mL), which engages gp130 and IL-6Rα, but not WSX-1, to induce STAT3 phosphorylation (Figure 4B) (17). These results showed clear IL-6-induced STAT3 phosphorylation as well as inhibition of this STAT3 phosphorylation by gp130 nAb treatment compared to mIgG1 isotype control. As expected, the WSX-1 Fc chimera did not interfere with IL-6-induced STAT3 phosphorylation.

To further determine the receptor requirements for IL-27- and IL-30-induced functions, we examined IP-10 expression at 24 h under the same conditions as above. IL-27-induced IP-10 expression was significantly but only partially affected by gp130 nAb at all doses, while the WSX-1 Fc chimera had a dose-dependent inhibition of IL-27-induced IP-10 expression in THP-1 cells (Figure 4C). IL-30-induced IP-10 expression was significantly but also partially inhibited at all three doses of the gp130 nAb, while the WSX-1 Fc chimera resulted in complete abrogation of IL-30-induced IP-10 at all doses tested (Figure 4C). In primary monocytes, the gp130 nAb and the WSX-1 Fc chimera had a partial effect on IL-27-induced IP-10 production, but the effect of the gp130 nAb did not reach statistical significance (Figure 4D). Similarly, the gp130 nAb partially reduced IL-30-induced IP-10 production, whereas the WSX-1 Fc chimera completely eliminated IL-30-mediated chemokine production although the effect of the gp130 nAb did reach statistical significance in primary monocytes (Figure 4D). These findings suggest that IL-30 signaling requires engagement of WSX-1 to induce cytokine production and gp130 is partially required.

### IL-30 Induces Significant Phosphorylation of STAT3 and STAT1 at Late Time Points

**Figure 5:**
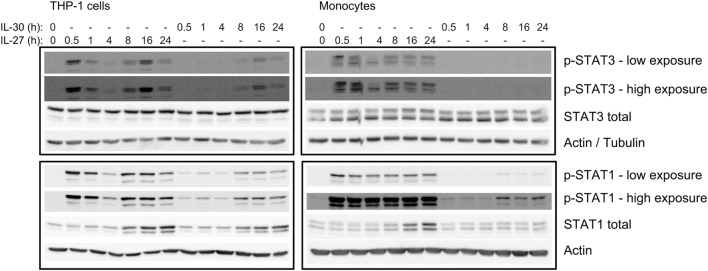
IL-30-induced phosphorylation of STAT3 and STAT1 is delayed. THP-1 cells and primary human monocytes were treated with or without recombinant IL-27 (50 ng/mL) or IL-30 (50 ng/mL) for 0.5–24 h as indicated. Phosphorylation of STAT3 and STAT1 and total STAT3 and STAT1 was measured by immunoblot, and β-actin was used as a loading control. Data are representative of three different THP-1 experiments or three different monocyte donors.

Since we observed that IL-30-induced IP-10 expression after 24 h was dependent on STAT3 (Figures 3B,C), we tested if IL-30 stimulation resulted in STAT3 phosphorylation at later time points. Thus, we stimulated THP-1 cells and primary monocytes with IL-30 or IL-27 for 0.5, 1, 4, 8, 16, or 24 h and measured phosphorylation of STAT3 and STAT1 (Figure 5). As previously shown, IL-27 significantly activated both STAT3 and STAT1 after 30 min, which decreased by 4-h stimulation in both cell types. Interestingly, IL-27 also induced a secondary wave of STAT signaling initiated at 8 h, peaking at 16 h, and decreasing at 24 h in both cell types. As well, stimulation with IL-27 increased total STAT1 expression in both cell types. As in Figure 3A, IL-30 did not significantly phosphorylate STAT3 at early time points, but after 16–24 h, STAT3 and STAT1 phosphorylation was observed in IL-30-treated THP-1 cells. In primary monocytes, IL-30-induced STAT3 phosphorylation was barely detectable at all time points, while STAT1 activation peaked after 8 h. Like IL-27, IL-30 increased total STAT1 expression at 8–24 h in both cell types, with a weaker IL-30-induced STAT1 expression observed in primary monocytes compared to the more robust response in THP-1 cells. Taken together, the data indicate that both IL-30 and IL-27 modulate STAT1 expression and suggest the existence of an intermediary interaction/protein required for downstream STAT1/3 activation and IP-10 production.

### sIL-6Rα Mediates IL-30- and IL-27-Induced IP-10 Production

**Figure 6:**
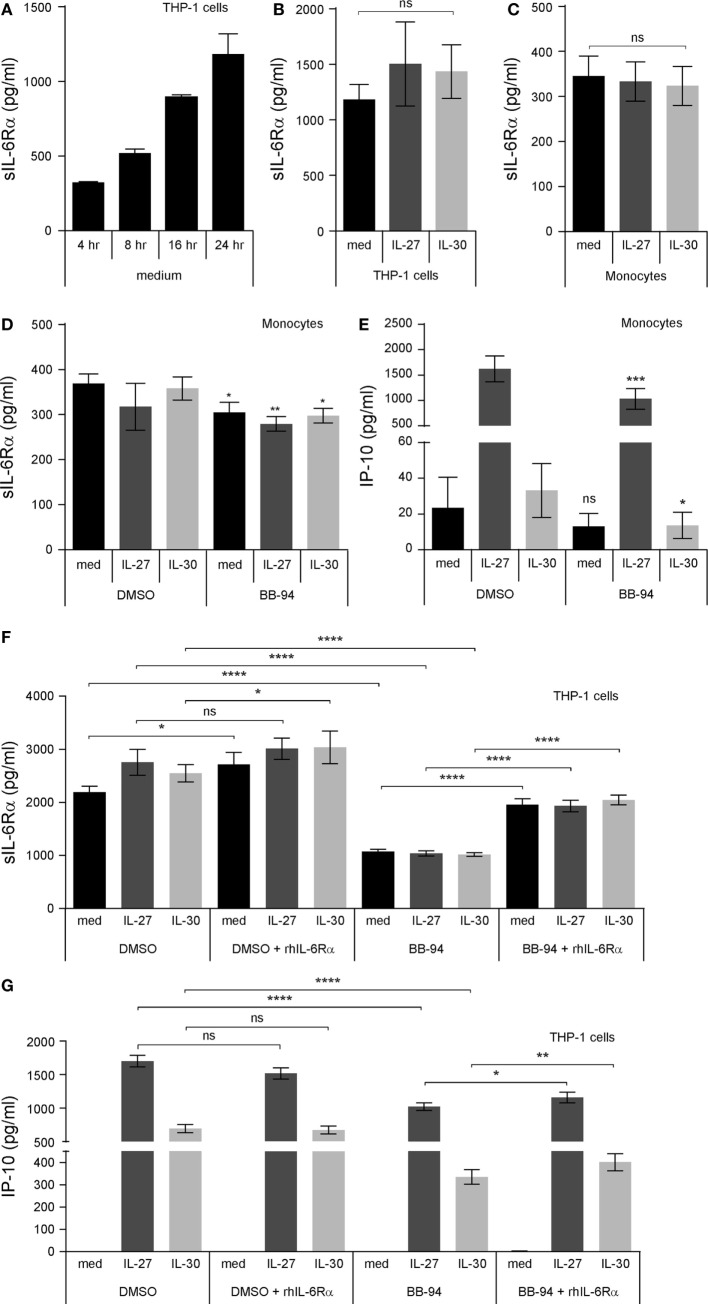
Cleaved soluble IL-6 receptor α (sIL-6Rα) is involved IL-27 and IL-30 function. **(A)** THP-1 cells were cultured in medium (RPMI + 10% FBS) for 4, 8, 16, and 24 h. Endogenously produced sIL-6Rα was measured in cell-free supernatants by ELISA. Data presented are the mean ± SEM of three different THP-1 experiments. **(B)** THP-1 cells and **(C)** primary human monocytes were treated with or without recombinant IL-27 (50 ng/mL) or IL-30 (50 ng/mL) for 24 h, and sIL-6Rα was measured in cell-free supernatants by ELISA. **(D,E)** Primary human monocytes were pretreated with BB-94 (25 µM) for 30 min and then treated with or without recombinant IL-27 (50 ng/mL) or IL-30 (50 ng/mL) for 24 h. **(D)** sIL-6Rα production and **(E)** IP-10 production were measured in cell-free supernatants by ELISA. **(F,G)** Recombinant human IL-6Rα was preincubated with IL-27 or IL-30 for 2 h. THP-1 cells were pretreated with BB-94 (25 µM) for 1 h and then stimulated with preincubated IL-6Rα (2.5 ng/mL) with or without IL-27 (50 ng/mL) or IL-30 (50 ng/mL) for 24 h. Pretreatment with DMSO was used as a vehicle control for BB-94. **(F)** sIL-6Rα production, and **(G)** IP-10 production was measured in cell-free supernatants by ELISA. Data presented are the mean ± SEM of at least three different THP-1 experiments or three different monocyte donors. Mann–Whitney *U* tests were used for statistical analyses between indicated groups **(B,C)**, between corresponding controls (DMSO) and BB-94 treatment **(D,E)**, or between pairs as indicated **(F,G)**, unless otherwise specified. Wilcoxon signed-rank matched pair tests were used for statistical analyses between medium or IL-30 control (DMSO) and BB-94 treatment **(E)**, between medium, IL-27, or IL-30 DMSO and DMSO + rhIL-6Rα treatment **(F)**, and between IL-27 or IL-30 BB-94 and BB-94 + rhIL-6Rα treatment **(G)**. ns, not significant; *p ≤ 0.05; **p ≤ 0.01; ***p ≤ 0.001; ****p ≤ 0.0001.

Interleukin-30 binds the sIL-6Rα subunit to signal via gp130 homodimers in murine cells (3, 11). In turn, we hypothesized that IL-30 may require sIL-6Rα for signaling in human cells. Increasing levels of sIL-6Rα were observed in supernatants upon culturing THP-1 cells for 4–24 h, indicating that culture conditions alone are sufficient to induce sIL-6Rα production in monocytic cells (Figure 6A). Next, we examined whether stimulation with IL-30 or IL-27 could induce upregulation of sIL-6Rα. Upon examination of time course stimulations for 4, 8, or 16 h, neither IL-30 nor IL-27 modulated sIL-6Rα levels (data not shown). Likewise, we found that there was no significant difference in sIL-6Rα production among medium-, IL-30-, or IL-27-treated monocytic cells after 24 h (Figures 6B,C). To determine if cleaved sIL-6Rα is involved in IL-30- and IL-27-induced IP-10 production, the matrix metalloproteinase inhibitor, BB-94 (batimastat), was added to the culture conditions to prevent cleavage of membrane bound IL-6Rα. Cells were treated with BB-94 for 30 min prior to IL-30 or IL-27 treatment for 24 h. In primary monocytes and THP-1 cells, BB-94 significantly reduced the formation of sIL-6Rα after 24 h compared to DMSO solvent controls (Figures 6D,F). Interestingly, THP-1 cells exhibited a greater decrease in sIL-6Rα expression in response to BB-94 compared to primary monocytes. This was recapitulated in the levels of IP-10 production induced by IL-30 and IL-27 in THP-1 cells and primary monocytes treated with BB-94 (Figures 6E,G). In light of the more prominent BB-94-mediated inhibition of sIL-6Rα secretion in THP-1 cells compared to primary monocytes, further investigation utilizing exogenous addition of recombinant sIL-6Rα was performed in THP-1 cells. For these experiments, IL-30 and IL-27 were preincubated for 2 h with recombinant sIL-6Rα at 2.5 ng/mL, as this concentration is in the range detected by ELISA in THP-1 culture medium (Figure 6F). The cells were cultured with either BB-94 or DMSO in the presence or absence of IL-30 or IL-27 alone or IL-30 + sIL-6Rα or IL-27 + sIL-6Rα for 24 h. Exogenous addition of recombinant sIL-6Rα following BB-94 or DMSO treatment resulted in statistically significantly increased detection of sIL-6Rα in all conditions, except in IL-27-stimulated DMSO-treated cells, which exhibited a minor increase that did not reach statistical significance (*p* = 0.1309) (Figure 6F). Addition of IL-30 + sIL-6Rα or IL-27 + sIL-6Rα to DMSO-treated control cells did not impact IP-10 expression. However, we observed a moderate, yet significant, increase in IP-10 production upon addition of complexed IL-30 + sIL-6Rα or IL-27 + sIL-6Rα compared to IL-30 or IL-27 alone in BB-94 treated cells (Figure 6G).

### IL-6 Pretreatment Downregulates Membrane IL-6Rα and gp130 for Decreased IL-30- and IL-27-Induced IP-10 Production

**Figure 7:**
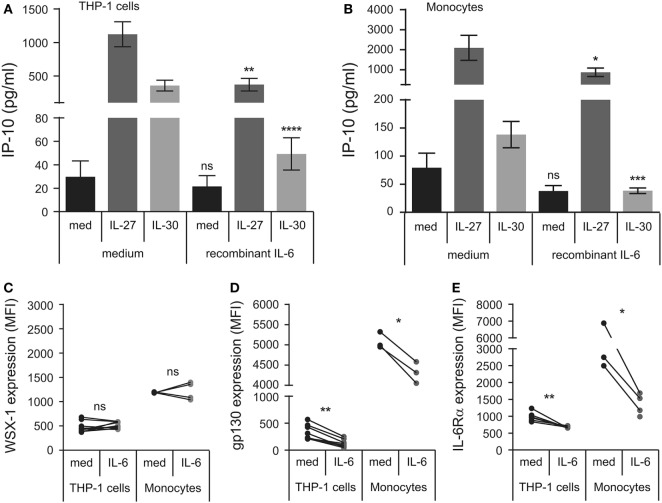
Recombinant IL-6 inhibits IL-27- and IL-30-IP-10 production. **(A)** THP-1 cells and **(B)** primary human monocytes were pretreated with recombinant IL-6 (10 ng/mL) for 30 min and then treated with or without recombinant IL-27 (50 ng/mL) or IL-30 (50 ng/mL) for 24 h. IP-10 production was measured in cell-free supernatants by ELISA. Data presented are the mean ± SEM of 13 different THP-1 experiments or 6 different monocyte donors. THP-1 cells and primary human monocytes were stimulated with or without recombinant IL-6 (10 ng/mL) for 30 min and then stained with IL-27 receptor α/T cell cytokine receptor (WSX-1) **(C)**, glycoprotein 130 (gp130) **(D)**, or anti-IL-6Rα **(E)**. Mann–Whitney *U* tests were used for statistical analyses between corresponding medium controls and IL-6 pretreated THP-1 cells. Wilcoxon signed-rank matched pair tests were used for statistical analyses between corresponding medium controls and IL-6 pretreated primary monocytes. ns, not significant; *p ≤ 0.05; **p ≤ 0.01; ***p ≤ 0.001; ****p ≤ 0.0001.

Interleukin (IL)-6 can signal *in trans* with sIL-6Rα and membrane-bound gp130 (17). To quench endogenous sIL-6Rα, we decided to test if delivery of recombinant IL-6 could modulate IL-30 or IL-27 responsiveness. Thus, we pretreated THP-1 cells and primary human monocytes with IL-6 for 30 min prior to the addition of IL-30 or IL-27 and measured IP-10 production after 24 h in cell-free supernatants (Figures 7A,B). IL-6 pretreated cells produced significantly less IL-27- and IL-30-induced IP-10 relative to cells that were not exposed to IL-6. Furthermore, we investigated the effect of IL-6 on membrane IL-6Rα, gp130, and WSX-1 in THP-1 cells and primary human monocytes after 30 min stimulation. IL-6 did not significantly alter WSX-1 expression in either cell type (Figure 7C). Alternatively, IL-6 significantly downregulated membrane-bound gp130 (Figure 7D) and IL-6Rα levels (Figure 7E) in both THP-1 cells and primary monocytes. Taken together, the data suggest that IL-30- and IL-27-induced IP-10 production is blocked by IL-6 as a result of internalization of IL-6Rα and/or gp130.

## Discussion

Interleukin (IL)-30, the p28 subunit of the heterodimeric cytokine IL-27, has been recently demonstrated to have biological activity in the absence of binding with EBI3 (3, 5, 11, 13). Our data indicate that in human monocytic cells, IL-30 shares overlapping pro-inflammatory functions with IL-27, but with reduced potency. The differential responsiveness between the two cytokines may be due to several factors. For example, IL-30 and IL-27 may exhibit varying affinities for gp130 and WSX-1. Furthermore, differences in protein folding of IL-27p28 when it is unbound or bound to EBI3 may also impede or enhance receptor binding, respectively. Differential responses to IL-30 and IL-27 were also observed between THP-1 cells and primary human monocytes, which may be attributed to a greater sensitivity of the primary cells compared to the cell line.

In our study, we used the same mass concentration of each cytokine [50 ng/mL; IL-27 (1 nM) and IL-30 (2 nM)]. To achieve the same molarity of IL-30 (IL-27p28 subunit), twice the mass concentration of IL-27 (100 ng/mL) is needed; however, given that IL-27 at a dose of 50 ng/mL was significantly more potent compared to 50 ng/mL of IL-30, we chose to keep the concentration (50 ng/mL) of each cytokine constant. This is in line with others who used IL-30 and IL-27 at the same mass concentration (18, 19). Consistent with our findings, Chong et al. demonstrated the biological activity of murine IL-30 in human cells (20). Furthermore, human and murine p28 amino acid sequences are 73% identical (1). In addition, we treated THP-1 cells with commercially available human IL-30 (Abnova) and observed no responses (data not shown), in agreement with others (19, 21); we attribute this to the presence of a GFP tag resulting in incorrect protein folding and hence a lack of biological activity (22, 23).

Interleukin (IL)-27 is well characterized to induce STAT3 and STAT1 phosphorylation in human immune cells via WSX-1 and gp130 receptor engagement (2, 24–31). In comparison, conflicting reports on IL-30-mediated signaling exist (3, 5), and likely the model and cell type explain these discrepancies. For instance, Crabé et al. demonstrated that p28/CLF-1 signals via STAT3 and STAT1 in Ba/F3 cells (3). In contrast, Stumhofer et al. determined that IL-30-stimulated murine T cells do not induce STAT3 or STAT1 phosphorylation and that IL-30 may function as a gp130 antagonist (5). In agreement with this, Chong et al. also demonstrated that IL-30 inhibited IL-27-induced STAT phosphorylation in human T cells and suggested that this occurs through IL-30 interaction with gp130 to antagonize the signaling pathway (20). We suggest that in human monocytes, IL-30 and IL-27 may depend on WSX-1 but may only partially require gp130. Our data demonstrating that the gp130 nAb abrogated IL-6-induced STAT3 rules out the possibility that the biological activity of the gp130 nAb was inefficient. Interestingly, we show that IL-27 and IL-30 upregulate total STAT1 expression after 8, 16, and 24 h. This may potentiate a greater availability of STAT1 for activation required for downstream IP-10 production and may provide a mechanism for the observed increase in STAT1 phosphorylation by IL-27 and IL-30 in our model. The late peak of IL-30-induced STAT3 phosphorylation, at 8–16 h posttreatment, in human monocytic cells together with the requirement of STAT3 for IL-30-mediated IP-10 expression, as seen using the STAT3-specific inhibitor Stattic, suggest the involvement of STAT3 phosphorylation for IP-10 production in response to IL-30.

**Figure 8:**
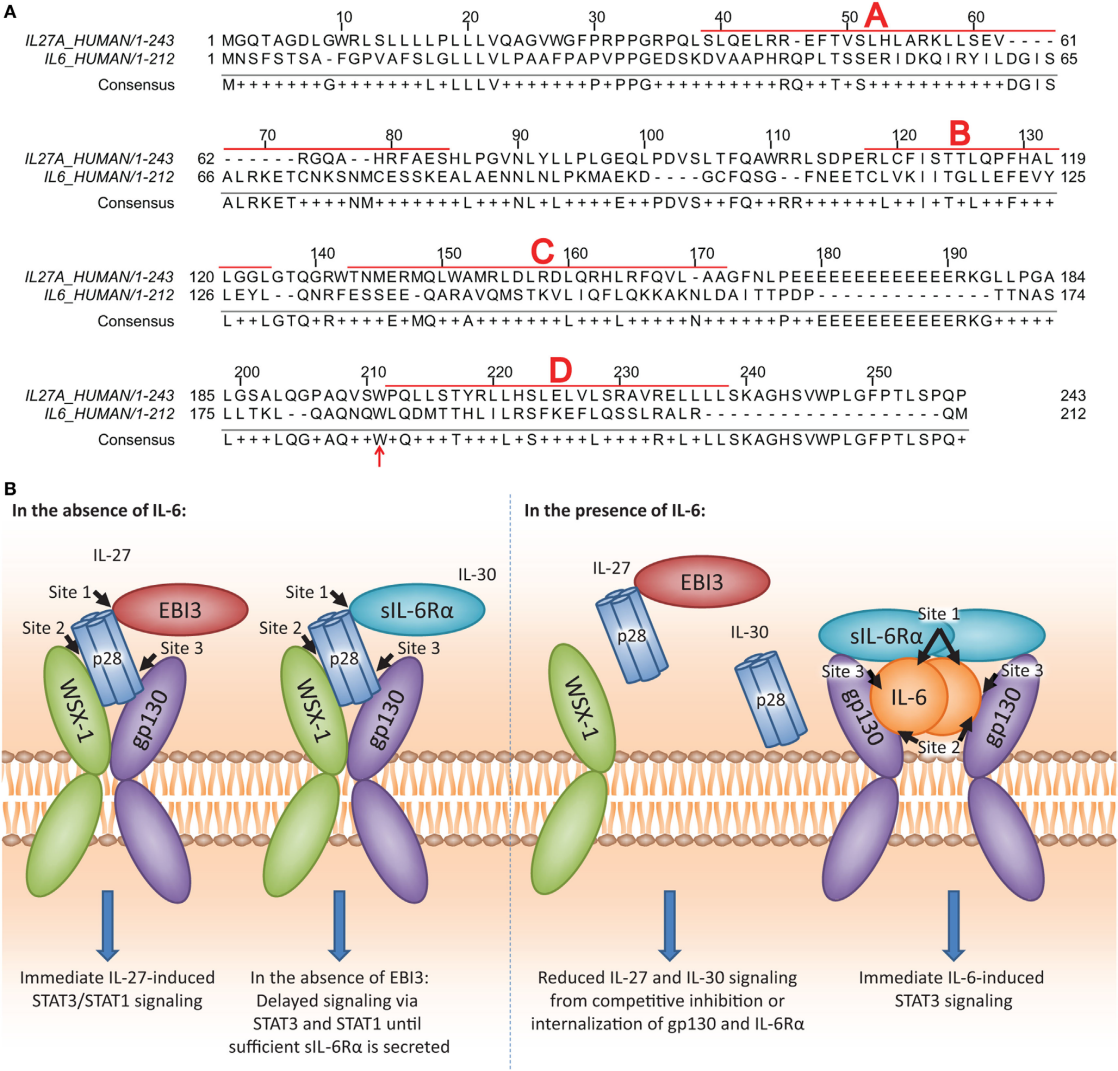
IL-27p28 and IL-6 sequence homology and proposed model of IL-30 and IL-27 interactions with IL-27 receptor α/T cell cytokine receptor (WSX-1), glycoprotein 130 (gp130), and soluble IL-6 receptor α (sIL-6Rα). **(A)** Human IL-27p28 (accession number: NP_663634.2) and human IL-6 (accession number: NP_000591.1) sequences were aligned using the T-COFFEE-expresso program (32). α-helices A, B, C, and D are indicated by bold red letters and boxed sequences. Conserved Trp^197^ on IL-27p28 and Trp^185^ on IL-6 are indicated by the red arrow. [**(B)**; left] The p28 subunit contains three binding sites (site 1, site 2, and site 3). For the binding of the dimeric IL-27, it has been demonstrated that the p28 site 1 interacts with Epstein–Barr virus-induced gene 3 (EBI3), site 2 interacts with WSX-1, and site 3 interacts with gp130 (36). For the binding of IL-30 (IL-27p28) to the WSX-1 and gp130 receptor chains, sIL-6Rα may be required. Since sIL-6Rα interacts with site 1 on IL-6, and IL-6, and IL-30 share homology, the p28 site 2 may interact with sIL-6Rα to enable IL-30-mediated signaling. Furthermore, sIL-6Rα may serve as an alternative binding partner in the absence of EBI3 to induce IL-30 signaling. [**(B)**; right] IL-6 may compete with IL-30 for interaction with sIL-6Rα due to higher affinity for IL-6. As a result, in the presence of IL-6, both IL-30 and IL-27 signaling and downstream functions are inhibited.

It is possible that similarities between IL-6 and IL-30 may govern the ability of IL-30, and therefore, the p28 subunit of IL-27, to interact with gp130 and/or IL-6Rα. Examination of amino acid similarity by T-COFFEE alignment software (32) revealed that IL-30 and IL-6 share 26.8% homology, in agreement with Gorshkova et al. (33) (Figure 8A). Furthermore, interactions between IL-6 and gp130/IL-6Rα have been well characterized, and three binding sites (site 1, site 2, site 3) on IL-6 are critical to this interaction (34, 35). IL-30 and IL-6 share a conserved tryptophan residue, Trp^197^ and Trp^185^, respectively, located in binding site 3 and is required for binding gp130 (Figure 8A; red arrow). IL-6Rα associates with IL-6 at site 1, and when complexed, IL-6/IL-6Rα associate with gp130 at IL-6 site 2 (34, 35). It has been noted that WSX-1 engages binding site 2 on IL-30 (36) although the specific amino acid(s) involved were not investigated. The Trp^97^ in binding site 1 is required for IL-30/EBI3 interaction and is conserved with dimeric IL-6 family members ciliary neurotrophic factor, neuropoietin, and cardiotrophin-like cytokine, although not with IL-6 (36), as this residue is required for heterodimeric cytokine interaction.

Early studies indicated that IL-30 forms a tripartite complex with the cytokine receptor subunit CLF-1 and/or sIL-6Rα (3, 5, 11). In agreement with these studies, we propose that IL-30 also interacts with sIL-6Rα in human monocytic cells (Figure 8B; left). Since IL-6 exhibits a greater affinity for IL-6Rα compared to IL-30 (11), and we show that IL-6 downregulates its receptor subunits immediately after binding, it is possible that IL-6 outcompetes IL-30 or IL-27 for IL-6Rα and gp130 engagement (Figure 8B; right). Given that sIL-6Rα and IL-6 complex for trans-signaling with membrane bound gp130 (37–39), and sgp130 functions to inhibit this signaling (40–42), it follows that these soluble cytokine receptors may also modulate IL-30 and IL-27 function. Treatment with BB-94, shown to inhibit sIL-6Rα formation (43), resulted in concurrent decreases in sIL-6Rα and IP-10 expression in both IL-30- and IL-27-treated cells. However, recombinant sIL-6Rα in complex with IL-30 or IL-27 induced only a moderate increase in IP-10 expression, suggesting that other molecules in addition to sIL-6Rα may be required for optimal IL-30 and IL-27 responses. Thus, although sgp130 does not inhibit IL-27 signaling (44), it is possible that endogenous sgp130 could associate with IL-30/IL-27 to enhance signaling via membrane bound WSX-1. Indeed, we observed slightly enhanced IL-27-induced STAT3 phosphorylation in the presence of gp130 nAb; this could be explained by inefficient binding of the nAb to sgp130, thus allowing IL-27 to complex with sgp130 for signaling through membrane WSX-1. Interestingly, we observed a range of IL-30 responsiveness in primary human monocytes, which could be attributed to differences in donor-specific expression levels of WSX-1 and soluble and membrane gp130/IL-6Rα. As well, BB-94 had a lesser effect on secreted sIL-6Rα levels in monocytes compared to THP-1 cells, raising the possibility that the majority of sIL-6Rα in cultured primary monocytes results from alternative splicing rather than cleavage of the membrane-bound form. IL-27-induced IP-10 production may result from direct IL-27-STAT signaling as evidenced by IP-10 production at the 4 h time point, whereas IL-30-induced IP-10 is evident after 8 h, suggesting that IL-30 signaling is delayed until sufficient levels of sIL-6Rα/sgp130 are released from the cell. Thus, a role for other soluble receptors such as sgp130 cannot be ruled out at this time, and further experimentation using platforms such as surface plasmon resonance is required to delineate relative binding affinities of IL-27 and IL-30 for IL-6Rα, gp130, and WSX-1.

Understanding cytokine signaling is vitally important in the study of innate immunity. Receptor engagement and JAK/STAT signaling in response to the novel cytokine IL-30 has not been clearly defined in human cells. In human monocytes, IL-27- and IL-30-induced IP-10 production and STAT phosphorylation requires WSX-1 engagement, while gp130 and sIL-6Rα are partially required. As IL-6 pretreatment inhibits IL-27- and IL-30-induced IP-10 production in human monocytic cells, we propose that IL-30 utilizes the endogenously produced soluble receptors such as IL-6Rα and gp130 to signal via WSX-1 in the absence of EBI3.

## Ethics Statement

This study was carried out in accordance with the recommendations of Canadian Tri-Council Policy Statement: Ethical Conduct for Research Involving Humans and Health Sciences and Affiliated Teaching Hospitals Research Ethics Board (HSREB) with written informed consent from all subjects. All subjects gave written informed consent in accordance with the Declaration of Helsinki. The protocol was approved by the HSREB.

## Author Contributions

CP and YY performed and analyzed experiments. MM performed and analyzed immunoblot assays. CP, NG, and KG designed the study and wrote the manuscript.

## Conflict of Interest Statement

The authors declare that the research was conducted in the absence of any commercial or financial relationships that could be construed as a potential conflict of interest.

## Abbreviations

EBI3, Epstein–Barr virus-induced gene 3; gp130, glycoprotein 130; IL, interleukin; IL-30, IL-27 p28 subunit; IP-10, interferon-γ-induced protein 10; sIL-6Rα, soluble IL-6 receptor α; WSX-1, IL-27 receptor α/T cell cytokine receptor.
